# Socialists and Hospitals

**Published:** 1888-11-24

**Authors:** William Moore

**Affiliations:** late Surgeon-General with the Government of Bombay


### Socialists and Hospitals.

By Sir William Moore, K.C.I.E., late Surgeon-General with the Government of Bombay.

"Whene'er I take my walks abroad, how many poor I see" was the observation of a poet now long since called to join the great majority. But the observation lives, and is likely to endure to the end of time; for we have it on the best authority that the poor shall always be with us. Other things are changed—opinions, manners, morals, creeds. Actions once esteemed without the pale are now venial errors. Opinions once regarded as scandalous are now the mark of advanced thought. Still, society in its two divisions of rich and poor—those who have more appetites than dinners, and those who have more dinners than appetites—rolls on for ever. Not only in Great Britain, but in every other country, the poor prevail, irrespective of climate, or race, or colour, or form of government. Unless the temperament, disposition, constitution, and habits of the great majority of mankind (and of woman-kind also) were strangely and impossibly altered, different circumstances of life must exist. If the Utopian schemes of advanced Socialism were practicable, if we all started to-morrow with similar advantages, the next day, even the next hour, would witness approach to inequality. One would gravitate towards loaferdom and *ignorance crasse*, another would saltate towards Midas-dom or philosophy. Even among the highest educated, temperament and constitution exert an imperial sway,

"For with like haste, in different ways they run,
Some to undo, and some to be undone."

A short time back, in a suburban locality, the writer accidentally encountered a gathering of people who, on tents, banners, and vans announced themselves as the "Socialist League." A Cassius-like personage, with "lean and hungry look," inflamed and reddened eyelids, weak chest, and spirituous-exhaling breath, hoped the writer approved of Socialism and was acquainted with the "Socialist's Catechism." Not receiving an answer in the affirmative, Cassius commenced an exhortation on the subject. He announced that Christianity and Socialism are one! The Socialists—at least his Socialists—did not prowl about like ravening wolves, seeking whom they might devour. They did not go about to commit murder, nor yet armed with guns and bombs, or swords and pistols. Nevertheless they intended to have their rights—if not by some means, then by other measures. What they wanted was equality, and the rights of man! They would have no West-end blades, going home at three o'clock in the morning. All should go to bed early, and rise, like the Whitechapelite, with the sun, in order to be fit and able for the day's work all ought to perform. As for kings and princes, there should be none of them—there was no such thing as Divine right, all were equal—adding irrelevantly, "A man's a man for a' that!" Whether Cassius knew whom he was quoting is perhaps *not* questionable. Tall talk of the kind, proceeding from that weak-chested, would-be leader of men, brought ridiculously to mind the old Cavalier song anent one bucolic recruit "who, when the Roundheads cried out 'Charge!' charged right another way." But the best point in the oratory of the Cassius-like one was, when h struck his chest and exclaimed, "Look at me! Am I a man with a *physic?* Am I like you appear to be, thirty-six inches round the chest, perhaps? And why am I not a man like you, with a *physic* and thirty-six inches round the chest? Why, because I live in Whitechapel and work hard every day, while you live in the West End and never had any work to do" (in which assertion the Cassius-like one was lamentably mistaken). On it being mildly intimated that peradventure drink might have something to do with the lack of physique, the soft insinuation was abundantly scorned; but at last it came from never to—well, "hardly ever." On being confronted with the argument that if all men were equal to-day, differences would initiate to-morrow, Cassius said, "No! they would take care of that; laws would be passed!" On inquiring who would constitute all equal, if he objected to permit people to enjoy the fruits of their labours—men for instance who had made themselves—he gave vent to the extraordinary declaration that there were no such persons! On being reminded of the case of a successful soldier, a successful merchant, and a successful physician, Cassius declared the soldier without the pale, because the soldier arrived at success by killing. Notwithstanding Gordon, and Hedley Vicars, of whom Cassius had never heard, he was quite sure a soldier, whose trade was killing people, could not be a good man, and therefore was unworthy to enjoy even what he earned. Well, as the soldier usually obtains more of honour, glory, or fame than of filthy lucre, this was not quite so bad for the soldier as the reply received by Mr. Roebuck when he informed a rustic of the death of Arthur, Duke of Wellington. "I be main sorry for he," said the rustic, "but who weer he?" Cassius declared the merchant could not even be considered, as the merchant attained position and wealth from the sweat of other people's brows. Here Cassius unwittingly repeated a fifteenth century dictum of the "Wise Child," as told in the "Bibliotheque Bleue" library. The Emperor Adrian having asked the "Wise Child" numerous questions appertaining to the sun, the land, the water, men and women, at length queried, "What hope have merchants?" "To perish," replied the Wise Child, "for what they gain comes by fraud and cheating!" So we see how a generally unmerited class stigma adheres through centuries! Lastly, Cassius declared the doctor to be worst of all! For the doctor experimented on poor people in hospitals in order that rich people might pay for the experience gained out of hospitals! Being rather tired of the business, the writer ventured to remark that most of the views expressed were Utopian and Quixotic. Whereupon a bystander observed that foreign words were not understood by English working-men. Although probably, too polite to say so—for a rough politeness certainly ruled in this camp of Socialists—the bystander evidently regarded the words used as appertaining to the Billingsgate of the upper classes. As neither argument nor explanation produced more effect on the witless socialist mind than the blows of a baby on the hide of a pachyderm, the writer soon afterwards obtained release and came away, recollecting the anecdote of the two men who were disputing on some matter, when one used the word paradox. "What is a paradox?" queried the second. "Paradox, paradox, paradox!" replied the first. "Oh, what is the use of arguing with anyone who does not know what a paradox is?"

We must not, however, be too hard on this would-be leader of men. It is so easy for those who have money or lands to think the Constitution perfect, while those who have not, naturally sigh for alteration. As Becky Sharpe pathetically observed, "It is so much more difficult to be good when one is poor." Besides, there is the precept generally attributable to Lord Avanly, that it would be hard because a man wanted money he should therefore want everything else. Moreover, in any state of life it is difficult to arrive at the philosophy of content. Modern men are not like Diogenes, satisfied with asking one favour. Grattan's definition of a competence was, "a little more than one has;" and the desire to arrive at the haven of competence is a meritorious ambition. But Socialists want not only turkey, but truffles also—a laudable aspiration enough; but the turkey and truffles must be earned, and not taken from other people. British Socialists teach that they work harder than anyone else, and are worse treated, both of which assertions may be questioned. Even among white or European races, English working-men would not win the prize for either industry or frugality. Among Eastern races the mild Hindoo works harder, and John Chinaman beats them all. No doubt Socialists of the class referred to are fully impressed with the idea that "laws grind the poor, and rich men rule the law." It may even be admitted, there is the proverbial morsel of bread in the metaphorical sack of their various plaints—plaints, however, which are "dash'd and brew'd" with unintentional lies, the result of ignorance. For they really know no more about many of the subjects they expatiate upon than the companions of Ulysses, after filling their ears with wax, knew of the Syrens singing. To these Socialists, the effects of temperament, constitution, and heredity on the human race are sealed books. Yet these forces are at the roots of social grades. It is not our province to combat Socialism; but it is our province to defend medicine from the attacks of Socialists. A more mistaken notion, than that which the weak-eyed leader of men propounded with reference to doctors and hospitals, could not emanate even from the brain of a Socialist. There is nothing unusual in men falling out with what is done for their own good; but their treatment in hospitals should be the last thing with which they fall out. The assertion that patients in hospitals are experimented on for the benefit of patients out of hospitals is not in accordance with facts. Doubtless, physicians and surgeons gain experience in hospital, but that experience is equally at the service of rich or poor, Socialists or Conservatives. That is the plain truth; but even a plain truth may be so worried and mauled by fallacies as to get the worst of it. A lie that is half a truth is ever the blackest of lies. Hospital patients may rest assured that their treatment in hospitals is always conducted towards the main point—their cure. It seems astounding that such an assertion should be necessary, but the weak-chested one's statement of the case was reverently received, and evidently, among a certain class, demands denial.

